# Antidiabetic effects of *Andrographis paniculata* supplementation on biochemical parameters, inflammatory responses, and oxidative stress in canine diabetes

**DOI:** 10.3389/fphar.2023.1077228

**Published:** 2023-02-14

**Authors:** Namphung Suemanotham, Sataporn Phochantachinda, Duangthip Chatchaisak, Walasinee Sakcamduang, Anchana Chansawhang, Pornsiri Pitchakarn, Boonrat Chantong

**Affiliations:** ^1^ Department of Clinical Sciences and Public Health, Faculty of Veterinary Science, Mahidol University, Nakhon Pathom, Thailand; ^2^ Department of pathology, Faculty of Veterinary Science, Chulalongkorn University, Bangkok, Thailand; ^3^ The Center for Veterinary Diagnosis, Faculty of Veterinary Science, Mahidol University, Nakhon Pathom, Thailand; ^4^ Department of Biochemistry, Faculty of Medicine, Chiang Mai University, Chiang Mai, Thailand; ^5^ Department of Pre-Clinic and Applied Animal Science, Faculty of Veterinary Science, Mahidol University, Nakhon Pathom, Thailand

**Keywords:** canine diabetes, oxidative stress, inflammation, adverse effects, *A. paniculata* (Burm.f.) nees (acanthaceae)

## Abstract

**Introduction:** Diabetes mellitus is a common endocrine disorder that causes hyperglycemia in dogs. Persistent hyperglycemia can induce inflammation and oxidative stress. This study aimed to investigate the effects of *A. paniculata* (Burm.f.) Nees (Acanthaceae) (*A. paniculata*) on blood glucose, inflammation, and oxidative stress in canine diabetes. A total of 41 client-owned dogs (23 diabetic and 18 clinically healthy) were included in this double-blind, placebo-controlled trial.

**Methods:** The diabetic dogs were further divided into two treatments protocols: group 1 received *A. paniculata* extract capsules (50 mg/kg/day; *n* = 6) or received placebo for 90 days (*n* = 7); and group 2 received *A. paniculata* extract capsules (100 mg/kg/day; *n* = 6) or received a placebo for 180 days (*n* = 4). Blood and urine samples were collected every month. No significant differences in fasting blood glucose, fructosamine, interleukin-6, tumor necrosis factor-alpha, superoxide dismutase, and malondialdehyde levels were observed between the treatment and placebo groups (*p* > 0.05).

**Results and Discussion:** The levels of alanine aminotransferase, alkaline phosphatase, blood urea nitrogen, and creatinine were stable in the treatment groups. The blood glucose levels and concentrations of inflammatory and oxidative stress markers in the client-owned diabetic dogs were not altered by *A. paniculata* supplementation. Furthermore, treatment with this extract did not have any adverse effects on the animals. Non-etheless, the effects of *A. paniculata* on canine diabetes must be appropriately evaluated using a proteomic approach and involving a wider variety of protein markers.

## 1 Introduction

Diabetes mellitus (DM) is a common endocrine disorder caused by absolute or relative insulin deficiency, which impairs glucose uptake into the cell (Nelson and Reusch, 2014). Insulin-dependent DM, a disease that resembles type 1 diabetes in humans, has been commonly recognized in dogs (Behrend et al., 2018). The diagnosis of DM in dogs is based on the presence of hyperglycemia and glucosuria, along with signs of weight loss, decreased appetite, polyuria, and polydipsia (Nelson and Reusch, 2014). Uncontrolled DM results in persistent hyperglycemia that can cause complications, such as diabetic cardiomyopathy, diabetic neuropathy, diabetic retinopathy, diabetic nephropathy, and atherosclerosis (Papatheodorou et al., 2016). The overproduction of superoxide has been proposed as a unifying mechanism that mediates the tissue-damaging effects of prolonged hyperglycemia (Brownlee, 2005). Thus, the management of DM is aimed at controlling the blood glucose level, which can be accomplished through insulin therapy, dietary modification, and control of concurrent disorders (Papachristoforou et al., 2020).

In combination with standard treatment, botanical drugs are utilized as an adjunct therapy to prevent long-term complications and improve the overall wellbeing of diabetic dogs. *A. paniculata* (Burm.f.) Nees (Acanthaceae) (*A. paniculate*), one of the most popular medicinal plants, consists of many active phytochemicals, such as andrographolide, neoandrographolide, andrographiside 14-deoxyandrographolide, 14-deoxy-11,12-didehydroandrographolide, 14-deoxy-11-oxoandrographolide, and β-sitosterol (Dai et al., 2019). The aerial part of this plant was commonly used due to the presence of andrographolide, the primary active component of *A. paniculata* (Patil and Andrographolide, 2020). The biological activities of andrographolide include anti-inflammatory, antioxidant, antiangiogenic, antidiabetic, antifertility, antiviral, antibacterial, cardioprotective, nephroprotective, and hepatoprotective effects (Okhuarobo et al., 2014; Kishore et al., 2017; Zhang et al., 2021). *A. paniculata* is known to exert anti-inflammatory properties by down-regulating the levels of cyclooxygenase and proinflammatory cytokines such as tumor necrosis factor-alpha (TNF-α), interleukin-6 (IL-6), and interleukin-10 (IL-10) in human, rats, and mice (Abu-Ghefreh et al., 2009; Zhang et al., 2013; Jaiyesimi et al., 2020; Zeng et al., 2022).

Evidence suggests that *A. paniculata* might be considered as a promising candidate for the management of DM; for instance, oral *A. paniculata* supplementation was used as a hypoglycemic agent in streptozotocin-induced diabetic rats (Yu et al., 2003) and high-fat-fructose-fed rats (Nugroho et al., 2012). Andrographolide supplementation ameliorated renal mesangial cell proliferation and inflammation by inhibiting protein kinase B (Akt) and nuclear factor-κB (NF-κB) signaling (Ji et al., 2016). Treatment of streptozotocin-induced diabetic rats with *A. paniculata* reduced oxidative stress by increasing the superoxide dismutase (SOD) and catalase activities (Zhang and Tan, 2000). Furthermore, decreased levels of malondialdehyde (MDA) and increased levels of glutathione were observed in the kidneys of diabetic rats supplemented with *A. paniculata* (Hidayat and Wulandari, 2021).

Studies in humans and rodents demonstrated the potential of *A. paniculata* as a supplemental treatment for DM in tandem with standard medicine. However, to the best of our knowledge, there is no published evidence of the impact and safety of *A. paniculata* supplementation in client-own diabetic dogs. Therefore, the aim of the present study was to evaluate the outcomes of *A. paniculata* supplementation in canine DM-associated inflammation and oxidative stress. Additionally, the adverse effects of *A. paniculata* supplementation were determined in the animals.

## 2 Materials and methods

### 2.1 Animals

**FIGURE 1:**
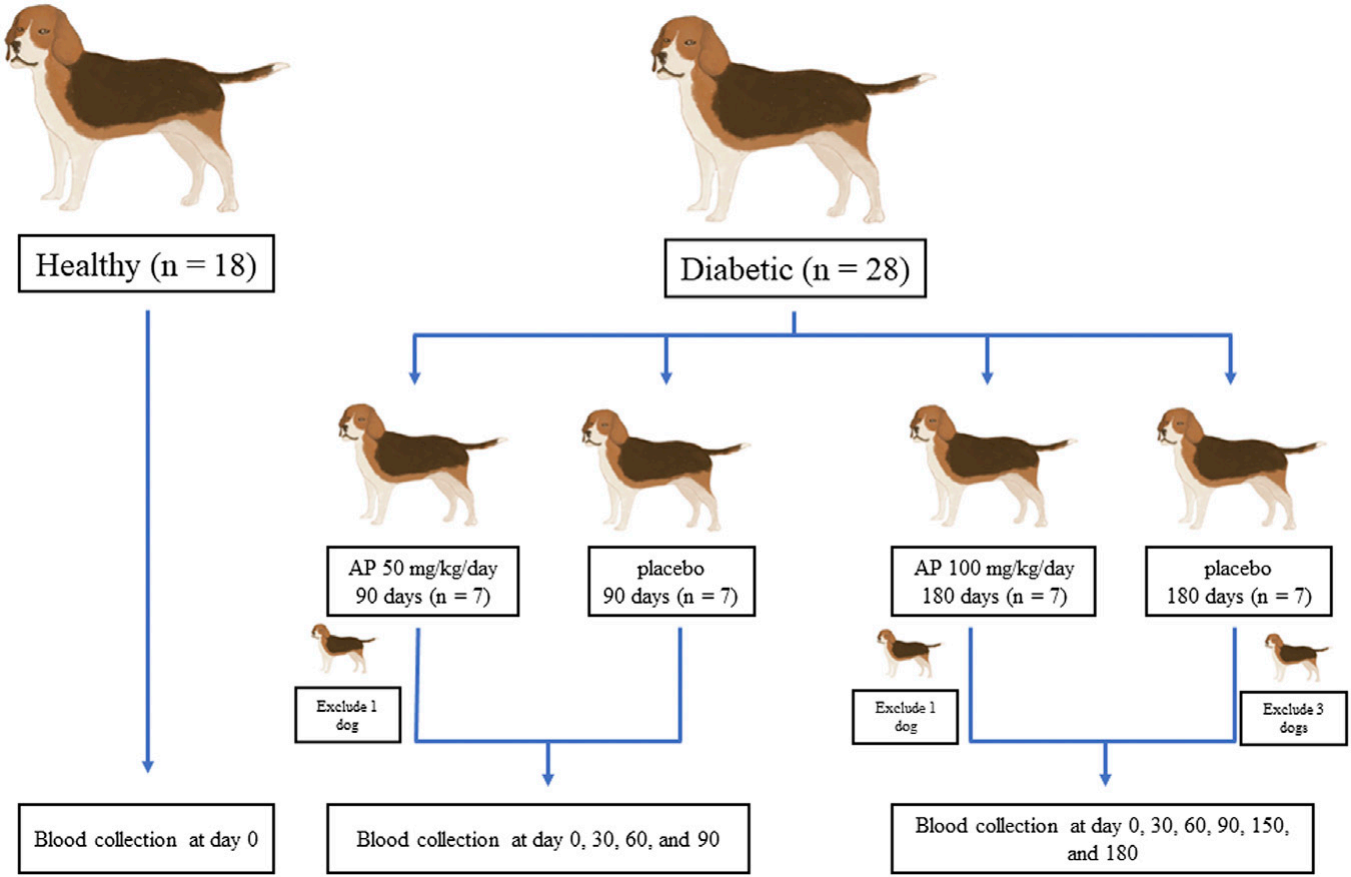
Design of experiments. Twenty-three diabetic and 18 healthy dogs were included in the study. The healthy dogs were used for cross-sectional baseline assessments. The diabetic dogs were divided into two treatment protocols to determine the appropriate dose and duration of *A. paniculata* administration. Dogs were given either *A. paniculata* extracted capsules (50 mg/kg/day; *n* = 6) or a placebo (*n* = 7) for 90 days. In another study, dogs were given *A. paniculata* extract capsules (100 mg/kg/day; *n* = 6), whereas others were given a placebo (*n* = 4) for 180 days.

This randomized, double-blind clinical trial was approved by the Committee for the Care and Use of Laboratory Animals, at the Faculty of Veterinary Science, Mahidol University (approval number: MUVS-2020-04-10). A total of 41 client-own dogs (23 diabetic and 18 clinically healthy) from Prasu Arthorn Animal Hospital, Faculty of Veterinary Science, Mahidol University were included in this study. The clinically healthy dogs were used for cross-sectional normal baseline evaluations. All dogs were used after obtaining the signed consent forms from the owners. The design used for the experiments in this study is shown in Figure 1. For the determination of optimum dose and duration of *A. paniculata* treatment, the diabetic dogs were divided into two treatment protocols. In the first protocol, dogs were given either *A. paniculata* extracted capsules (50 mg/kg/day; *n* = 6) or a placebo (*n* = 7) for 90 days. In another protocol, dogs were given *A. paniculata* extracted capsules (100 mg/kg/day; *n* = 6), while others were given placebo (*n* = 4) for 180 days. Routine treatment was provided to all the dogs. The clinical parameters and the levels of the inflammatory and oxidative stress biomarkers were evaluated in each group. The characteristics of the dogs in each group are shown in Supplementary Table S1A.

The inclusion criteria were diabetic dogs of any breed, age, or sex, with stable blood glucose levels for the previous 3 months. The diabetic dogs were diagnosed with a history of polyuria, polydipsia, polyphagia, weight loss with normal or increased appetite, fasting hyperglycemia, and glucosuria. The exclusion criteria for the study were as follows: dogs with unstable diabetes or diabetic ketoacidosis, those that received corticosteroids, and those with diseases that affect the blood glucose levels, such as hyperadrenocorticism, exocrine pancreatic insufficiency, neoplasia, and acromegaly. To reduce the confounding factors, all the diabetic dogs enrolled in this study were fed a commercial diabetic diet and allowed to live indoors or within their compounds, close to their owners, without any changes in their environment during the study period.

### 2.2 *A. paniculata* and placebo

A commercial *A. paniculata* capsule (Abhaibhubejhr^**®**^ FAH-TALAI-JONE capsule, lot: MB00916006) was used as a supplement in this study. Each capsule contained 400 mg of the aerial part of *A. paniculata*, which was standardized to a content of 1% w/w andrographolide. The procedures and findings of the analysis of andrographolide in the capsule of *A. paniculata* are detailed in Supplementary material B. Lactose powder, which was similar in appearance to the *A. paniculata* capsule, was used as a placebo in this study. The dosage of the drug was referenced from the dosage given in experimental rats in a previous study (Panossian et al., 2000) and calculated to that used for dogs using the conversion factors proposed by Reagan-Shaw (Reagan-Shaw et al., 2008).

### 2.3 Sample collection

Blood and urine samples were collected from clinically healthy and diabetic dogs on day 0 for the baseline data. The blood and urine samples from the diabetic dogs were reassessed every 30 days for 90 or 180 days, depending on the treatment protocol. Blood samples (3–5 mL) were collected from the cephalic or saphenous vein to evaluate the clinical parameters and the biomarkers for inflammation and oxidative stress. The clinical parameters, which included complete blood count (CBC) and the levels of glucose, fructosamine, alanine aminotransferase (ALT), alkaline phosphatase (ALP), blood urea nitrogen (BUN), and creatinine, were analyzed every 30 days during routine health check-up. IL-6 and TNF-α were selected as the inflammatory biomarkers, whereas SOD and MDA were used as the oxidative stress biomarkers. The biomarkers were evaluated on days 0 and 90 or 180, depending on the treatment protocol.

One drop of the blood sample was used to test the glucose level using the AlphaTRAK glucometer (Zoetis, Parsippany, NJ, United State). The blood samples were then divided into three parts and used for further experiments. The ethylenediaminetetraacetic acid (EDTA) blood samples were stored at 4°C, and the CBCs were measured using an animal blood counter (Horiba Medical, Montpellier, France) within 4 h. The second part was centrifuged at 3500 rpm for 5 min within an hour after blood collection; the resultant plasma was used to measure the ALT, ALP, BUN, and creatinine levels using an automatic analyzer (Chema diagnostica, Monsano AN, Italy), and the serum was collected in a sterile microcentrifuge tube and stored at −80°C to measure the IL-6, TNF-α, SOD, and MDA levels. The third part was collected in plain tubes and sent to a commercial laboratory within 24 h to measure the fructosamine level.

The urine sample was collected by cystocentesis and centrifuged at 2000 rpm for 3 min (Hettich Lab Technology, Tuttlingen, Germany). The physical and chemical properties (color, clarity, specific gravity, pH, protein, glucose, ketone, and bilirubin levels, and erythrocyte counts) of the urine supernatants were tested using the dipstick test (Roche Diagnostics, Indianapolis, IN, United State). The urine sediments were evaluated under a light microscope (ZEISS, Jena, Germany). The white blood cell counts (high-power field: HPF), red blood cell counts (HPF), and presence of amorphous crystals, mucous, bacteria, epithelium cells (HPF), cast (low-power field: LPF), and crystals were determined.

### 2.4 Interleukin-6 (IL-6) and tumor necrosis factor-α (TNF-α) determination

The levels of IL-6 and TNF-α in the plasma were measured by an enzyme-linked immunosorbent assay (ELISA) assay using kits obtained from Abcam (Cambridge, United Kingdom) and Thermo Scientific (Waltham, MA, United State), respectively. The procedure was carried out in accordance with the manufacturer’s instructions. An ELISA plate reader (BioTek, Santa Clara, CA, United State) was used to measure the absorbance at 450 nm, and the IL-6 and TNF-α levels were compared with those of the standards.

### 2.5 Superoxide dismutase (SOD) determination

The levels of SOD in the serum were measured by ELISA using commercial kits (Abcam, Cambridge, United Kingdom) according to the manufacturer’s protocols. The microplate reader (BioTek, Santa Clara, CA, United State) was used to read the optical density at 450 nm, and the SOD activity was calculated in percentage.

### 2.6 Malondialdehyde (MDA) determination

The levels of MDA in plasma were measured using the thiobarbituric acid reactive substance assay, which was based on the reaction between MDA and thiobarbituric acid; the reaction resulted in a pink-colored product, which demonstrated absorption at a wavelength of 535 nm (Gheita et al., 2014). Briefly, plasma samples (0.1 mL) were mixed with 1 mL of 6.7 mg/mL of orthophosphoric acid (Sigma, St. Louis, MO, United State) and 1 mL of 0.13% thiobarbituric acid (Sigma, St. Louis, MO, United State). The solution was thoroughly mixed and heated in a boiling water bath for 45 min; after cooling down, 0.8 mL of n-butanol was added to the solution and mixed vigorously. The n-butanol layer was separated by centrifugation at 3,000 rpm for 15 min (Beckman Coulter, Brea, CA, United State) and retained for spectrometric analysis. The absorbance of the pink-colored product within the butanol layer was measured with a microplate reader (BioTek, Santa Clara, CA, United State) at 535 nm and 520 nm for interference subtraction. The blank in this protocol contained distilled water. The concentrations of MDA were calculated from the standard MDA solution (range, 0.1–20 nmol/mL).

### 2.7 Statistical analysis

All data from the experiments were assessed for normal distribution using the Shapiro–Wilk test and for variance using Levene’s test. Data from the clinically healthy dogs were compared with those from the diabetic dogs prior to *A. paniculata* supplementation (day 0) using the independent *t*-test. The clinical parameters of DM were compared between day 0 and days 30, 60, 90, 120, 150, and 180 (depending on the treatment protocol) using repeated measures analysis of variance. The inflammatory and oxidative stress parameters were compared between day 0 and day 90 (treatment protocol 1) or day 0 and day 180 (treatment protocol 2) using the paired *t*-test. Statistical calculations were performed using SPSS version 21 (IBM, Armonk, NY, United State), and the significance level was set at *p* ≤ 0.05.

## 3 Results

### 3.1 Descriptive characteristics of the dogs at inclusion

**FIGURE 2:**
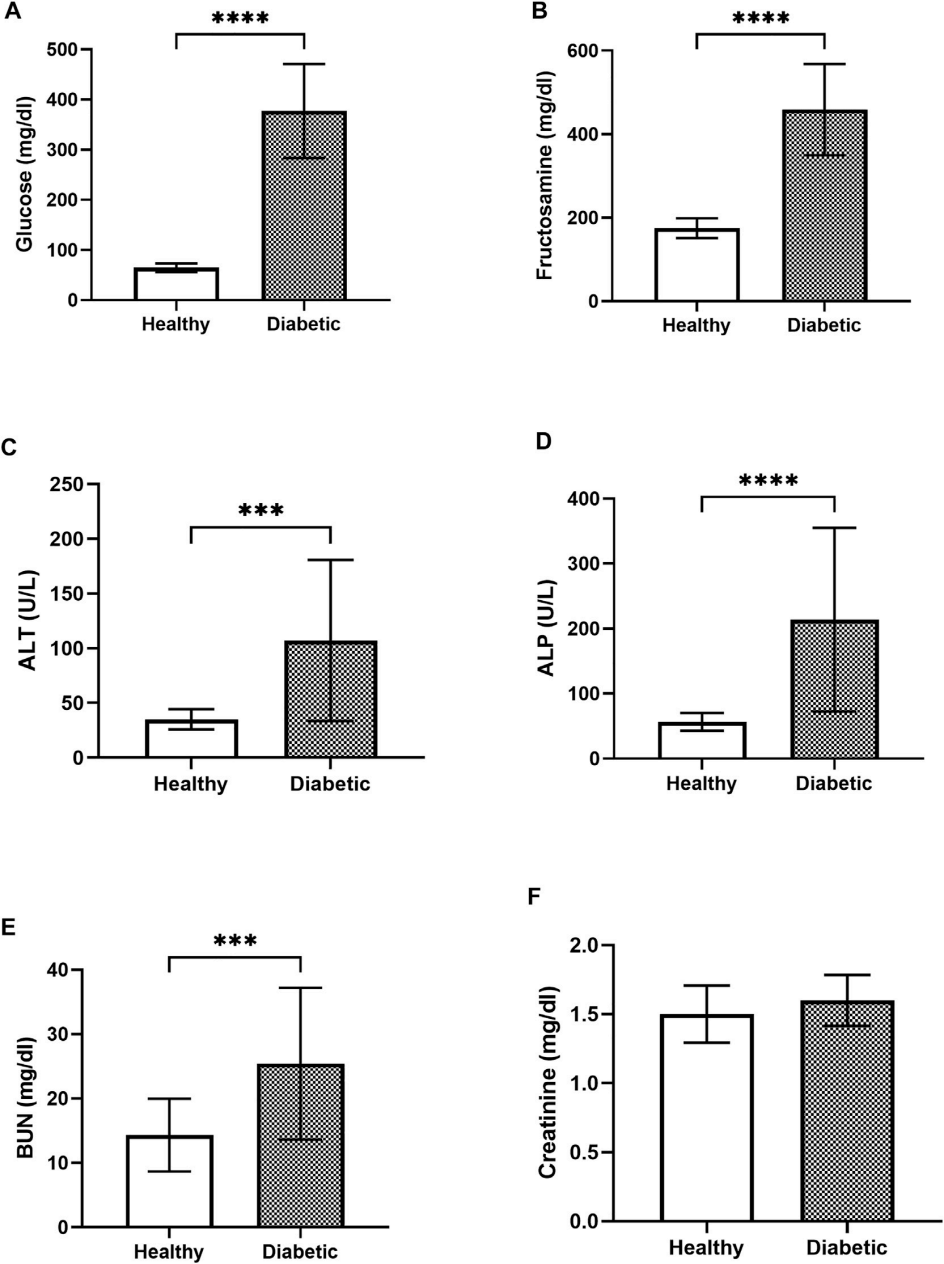
Bar graphs showing the blood glucose **(A)**, blood fructosamine **(B)**, ALT **(C)**, ALP **(D)**, BUN **(E)**, and creatinine **(F)** levels in the healthy and diabetic group of dogs **** = *p* < 0.0001, *** = *p* < 0.001.

The baseline parameters of the clinically healthy (*n* = 18) and the diabetic (*n* = 23) dogs prior to *A. paniculata* supplementation were evaluated to minimize bias between groups. The average fasting blood glucose level in the diabetic dogs (377.33 ± 91.83 mg/dL) was significantly (*p* < 0.01) higher than that in the clinically healthy dogs (64.67 ± 8.36 mg/dL; Figure 2A). Similarly, the average blood fructosamine level in the diabetic dogs (459 ± 159.42 umol/L) was significantly (*p* < 0.01) higher than that in the clinically healthy dogs (175 ± 23.22 umol/L; Figure 2B).

The blood biochemistry results showed that the ALT levels in the clinically healthy and the diabetic groups were 35 ± 8.99 U/L and 107.08 ± 72.07 U/L, respectively (Figure 2C). The measured ALP was 56.5 ± 13.3 U/L in the clinically healthy group and 213.86 ± 138.23 U/L in the diabetic group (Figure 2D). Likewise, the BUN levels in the clinically healthy and diabetic groups were 14.33 ± 5.50 U/L and 25.42 ± 11.54 U/L, respectively (Figure 2E), while those of creatinine were 1.5 ± 0.2 mg/dL and 1.6 ± 0.18 mg/dL, respectively (Figure 2F). The diabetic dogs had significantly higher levels of ALT, ALP, and BUN compared to the clinically healthy dogs (*p* < 0.05), whereas the creatinine levels did not differ significantly between the two groups.

The urine dipstick results showed that all the diabetic dogs, but none of the healthy dogs, had glucosuria. The urine samples from the diabetic and clinically healthy dogs were negative for ketone with inactive urine sediments (<5 white blood cells per HPF, <5 red blood cells per HPF, no bacteria, no epithelium cell, no cast, and no crystal).

**FIGURE 3:**
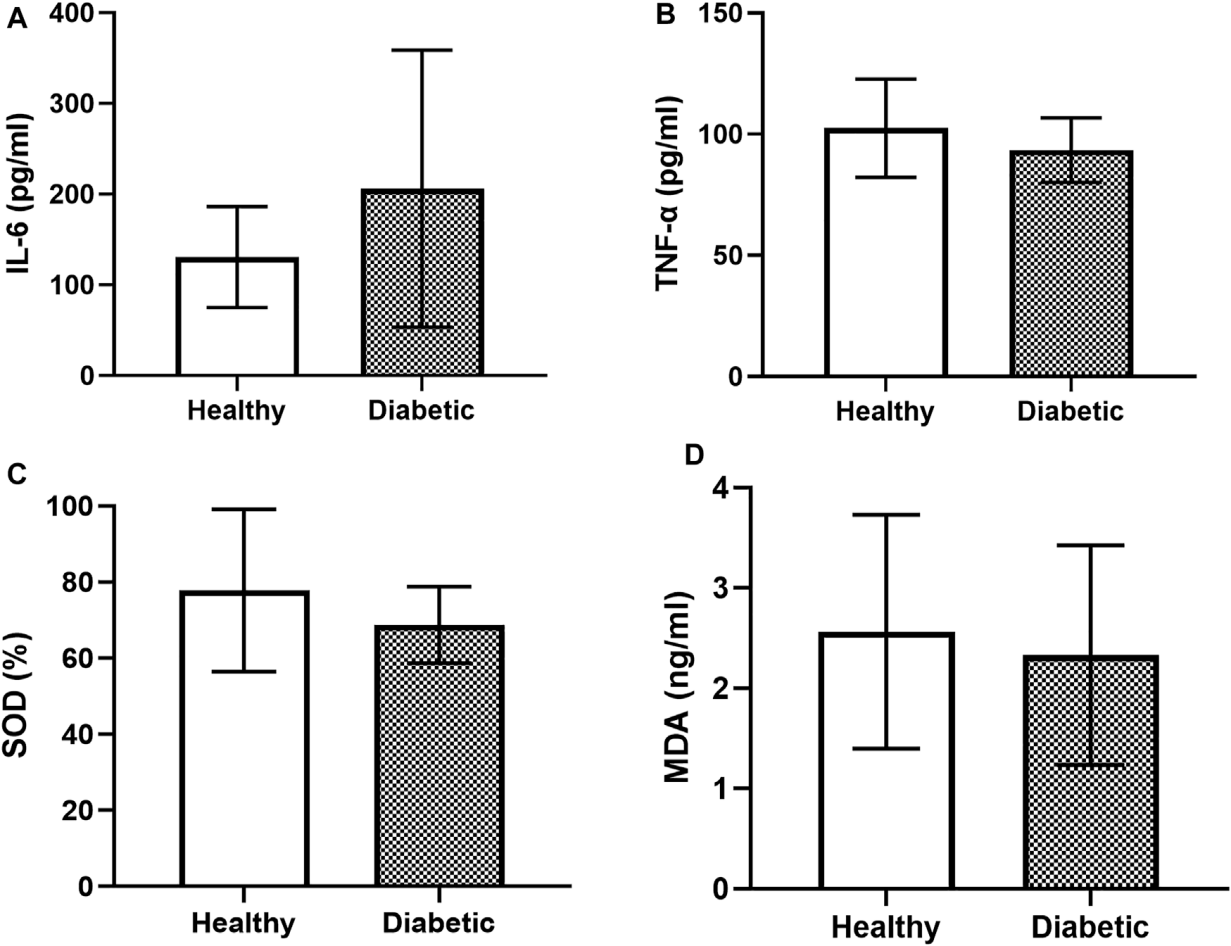
Bar graphs showing the baseline levels of the inflammatory and oxidative stress parameters in the clinically healthy and diabetic dogs **(A)** IL-6 **(B)** TNF-α **(C)** SOD, and **(D)** MDA.

The IL-6 levels in the healthy dogs (130.89% ± 54.06%) were not significantly different from those in the diabetic dogs (206.17% ± 149.32%; Figure 3A). The level of TNF-α in the healthy dogs (102.45% ± 19.64%) was slightly higher but not significantly different from that in the diabetic dogs (93.38% ± 13.05%; Figure 3B). Similarly, no significant differences in the levels of SOD (Figure 3C) and MDA (Figure 3D) were observed between the two groups.

### 3.2 Effect of *A. paniculata* supplementation on fasting blood glucose and fructosamine levels in the diabetic dogs

**FIGURE 4:**
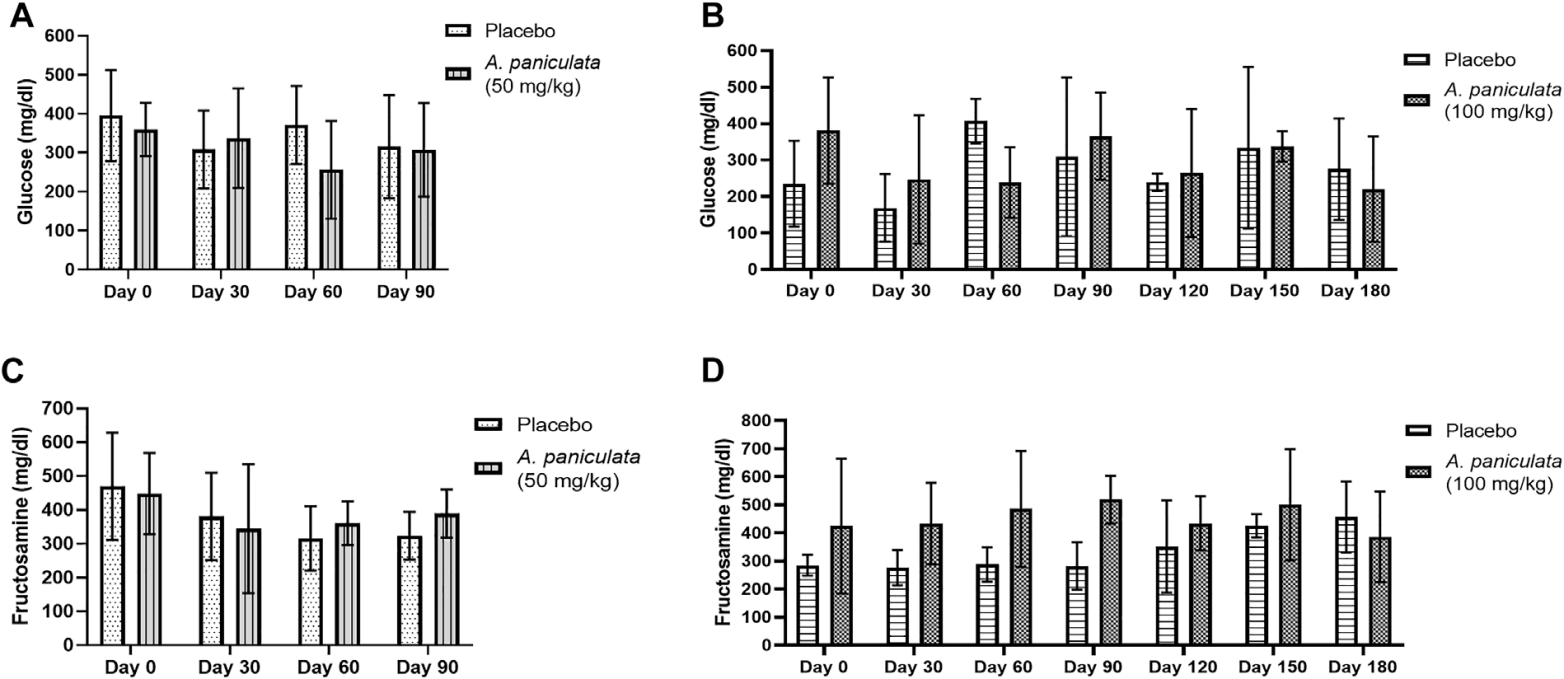
The effects of *A. paniculata* (dose 50 mg/kg/day for 90 days and 100 mg/kg/day for 180 days) on fasting blood glucose and blood fructosamine levels. Bar graphs showing blood glucose levels in groups treated with 50 mg/kg of *A. paniculata* and placebo for 90 days **(A)**, blood glucose levels in groups treated with 100 mg/kg of *A. paniculata* and placebo for 180 days **(B)**, blood fructosamine levels in groups treated with 50 mg/kg of *A. paniculata* and placebo for 90 days **(C)**, and blood fructosamine levels in groups treated with 100 mg/kg of *A. paniculata* and placebo for 180 days **(D)**.

The effects of *A. paniculata* (dose 50 mg/kg/day for 90 days and 100 mg/kg/day for 180 days) on fasting blood glucose and blood fructosamine levels were investigated. The fasting blood glucose (Figure 4A) and fructosamine (Figure 4C) levels in diabetic dogs supplemented with 50 mg/kg/day of *A. paniculata* for 90 days were not significantly lower than those in the placebo groups. Similarly, supplementation with 100 mg/kg/day of *A. paniculata* for 180 days did not alter the fasting blood glucose (Figure 4B) and fructosamine (Figure 4D) levels in the diabetic group compared to those in the placebo groups.

### 3.3 Effect of *A. paniculata* supplementation on inflammation in diabetic dogs

**FIGURE 5:**
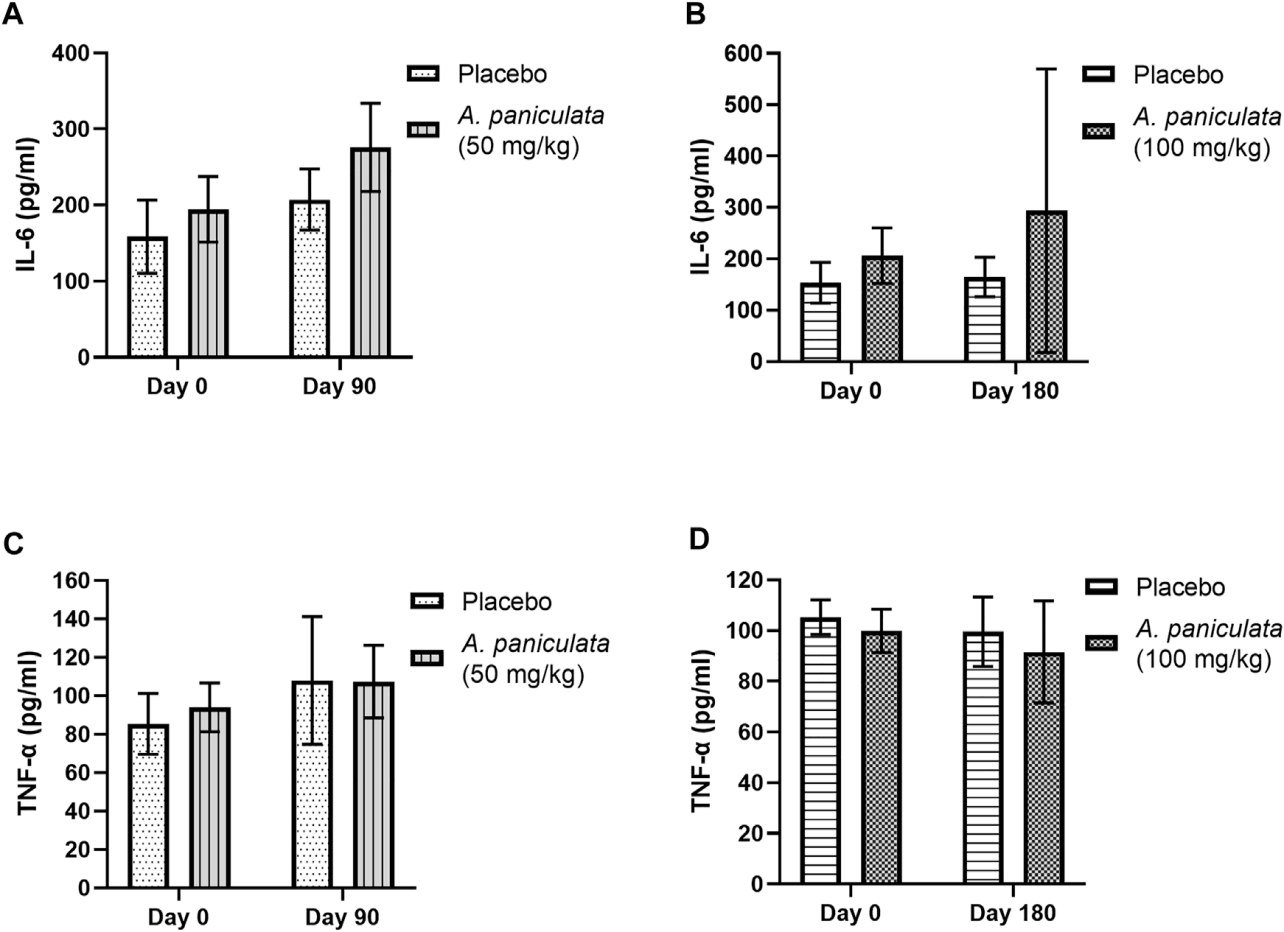
The effects of *A. paniculata* supplementation (doses 50 and 100 mg/kg) on inflammation in the diabetic dogs. Bar graphs showing no significant differences in the **(A)** IL-6 levels of dogs treated with 50 mg/kg of *A. paniculata* for 90 days, **(B)** IL-6 levels of diabetic dogs treated with 100 mg/kg of *A. paniculata* for 180 days, **(C)** TNF-α levels of diabetic dogs treated with 50 mg/kg of *A. paniculata* for 90 days, and **(D)** TNF-α levels of diabetic dogs treated with 100 mg/kg of *A. paniculata* for 180 days compared to those in the corresponding placebo groups.

The outcomes of *A. paniculata* (dose 50 mg/kg/day for 90 days and 100 mg/kg/day for 180 days) on inflammation were investigated. Supplementation with 50 and 100 mg/kg/day for 90 and 180 days, respectively, did not significantly alter the levels of IL-6 and TNF-α in the diabetic dogs when compared to those in the placebo groups (Figures 5A–D) levels.

### 3.4 Effect of *A. paniculata* supplementation on oxidative stress in diabetic dogs

**FIGURE 6:**
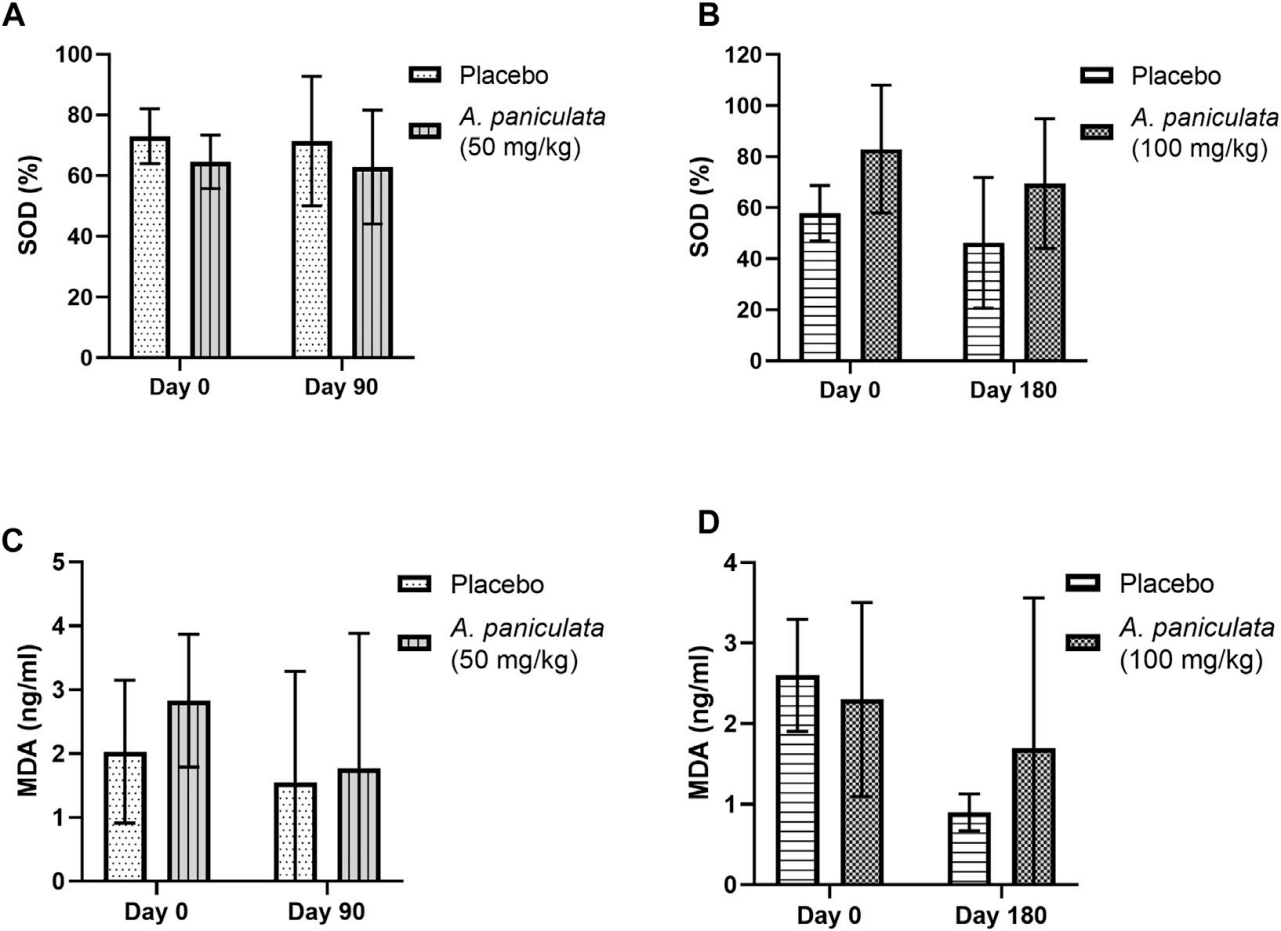
The effects of *A. paniculata* supplementation (doses 50 and 100 mg/kg for 90 and 180 days, respectively) on the oxidative stress parameters in the diabetic dogs. Bar graphs showing no significant differences in the **(A)** SOD levels of dogs treated with 50 mg/kg of *A. paniculata* for 90 days, **(B)** SOD levels of diabetic dogs treated with 100 mg/kg of *A. paniculata* for 180 days, **(C)** MDA levels of diabetic dogs treated with 50 mg/kg of *A. paniculata* for 90 days, and **(D)** MDA levels of diabetic dogs treated with 100 mg/kg of *A. paniculata* for 180 days compared to those in the corresponding placebo groups.

*A. paniculata* supplementation with 50 and 100 mg/kg/day for 90 and 180 days, respectively, did not significantly alter the SOD and MDA levels in the diabetic dogs compared to those in the placebo groups (Figures 6A–D).

### 3.5 Effect of *A. paniculata* supplementation on the biochemical parameters of liver injury and kidney function

**FIGURE 7:**
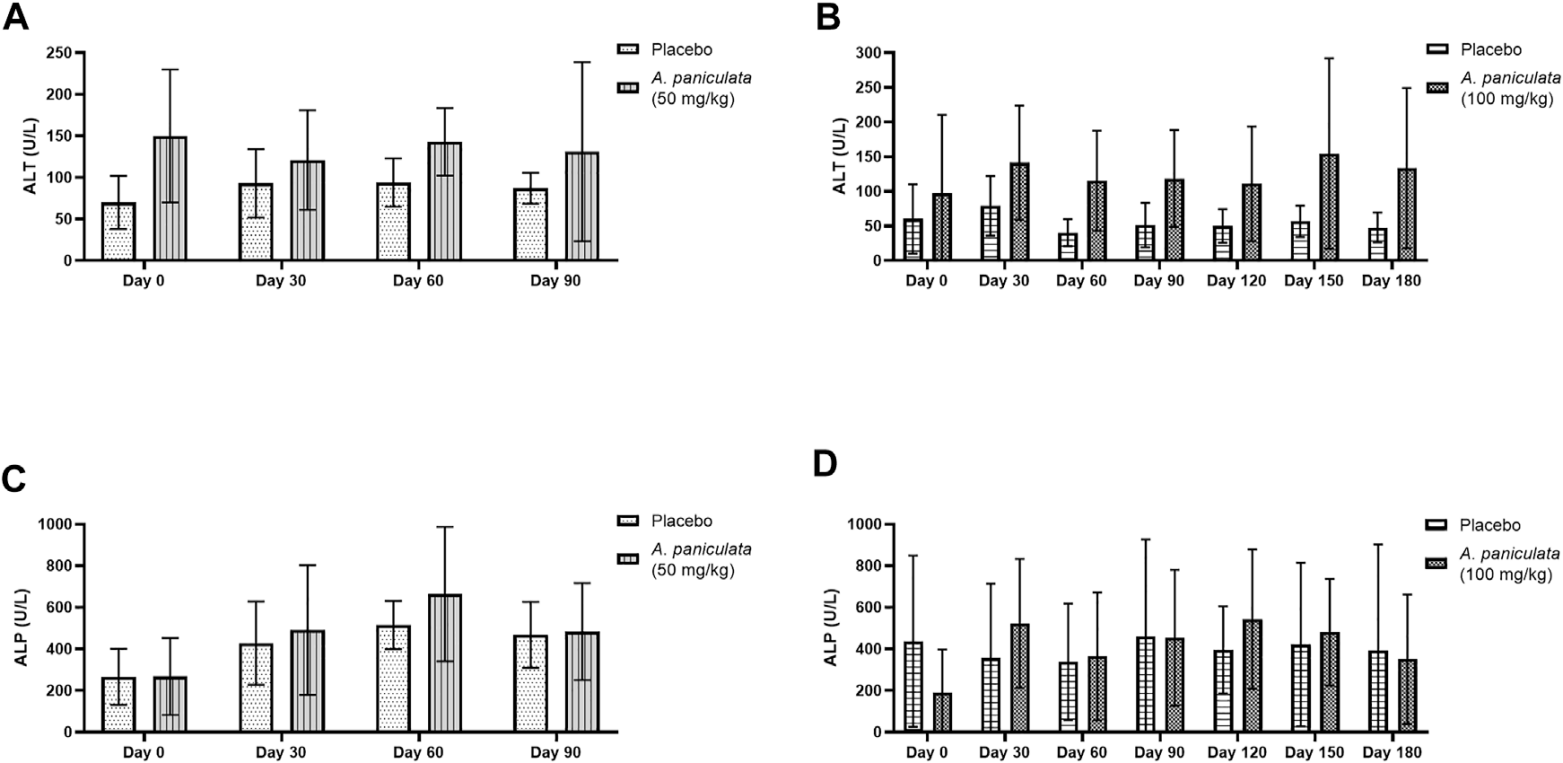
The effects of *A. paniculata* supplementation (doses 50 and 100 mg/kg for 90 and 180 days, respectively) on the biochemical parameters of liver injury in the diabetic dogs compared to those in the placebo groups. Bar graphs showing no significant differences in the **(A)** ALT levels of dogs treated with 50 mg/kg of *A. paniculata* for 90 days, **(B)** ALT levels of diabetic dogs treated with 100 mg/kg of *A. paniculata* for 180 days, **(C)** ALP levels of diabetic dogs treated with 50 mg/kg of *A. paniculata* for 90 days, and **(D)** ALP levels of diabetic dogs treated with 100 mg/kg of *A. paniculata* for 180 days compared to those in the corresponding placebo groups.

**FIGURE 8:**
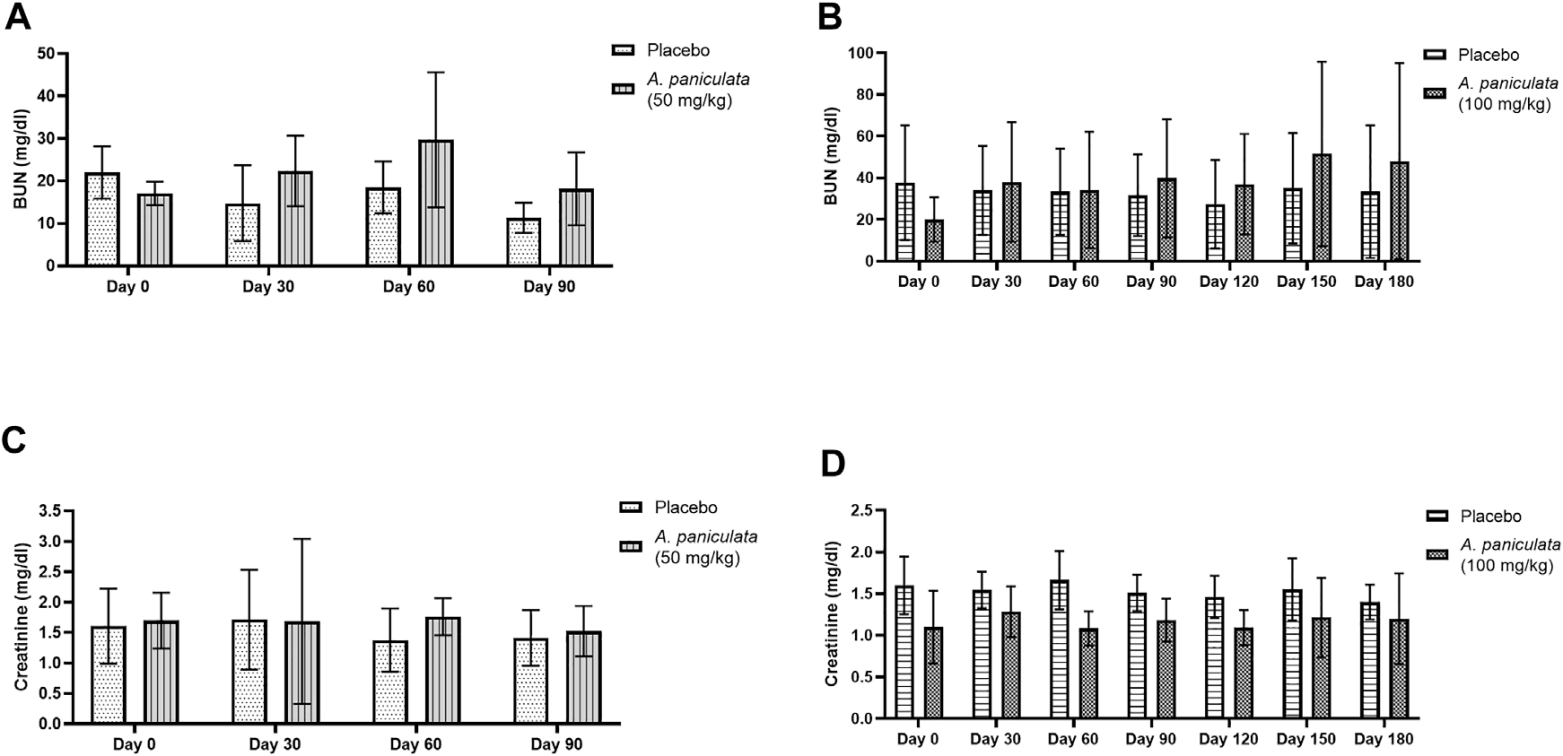
The effects of *A. paniculata* supplementation (doses 50 and 100 mg/kg for 90 and 180 days, respectively) on the biochemical parameters of renal injury in the diabetic dogs. Bar graphs showing no significant differences in the **(A)** BUN levels of dogs treated with 50 mg/kg of *A. paniculata* for 90 days, **(B)** BUN levels of diabetic dogs treated with 100 mg/kg of *A. paniculata* for 180 days, **(C)** creatinine levels of diabetic dogs treated with 50 mg/kg of *A. paniculata* for 90 days, and **(D)** creatinine levels of diabetic dogs treated with 100 mg/kg of *A. paniculata* for 180 days compared to those in the corresponding placebo groups.

Blood ALT and ALP were selected as the liver injury markers, and BUN and creatinine levels were used to examine the kidney function. No statistically significant changes in the levels of the liver injury markers (Figure 7) and kidney function markers (Figure 8) were noted after supplementation with *A. paniculata* (dose 50 mg/kg/day for 90 days and 100 mg/kg/day for 180 days) compared to those in the placebo groups.

The urinalysis results from day 0 to day 180 showed negative ketone bodies and inactive urine sediments (<5 white blood cells per HPF, <5 red blood cells per HPF, and no bacteria, epithelial cells, casts, and crystals), indicating the absence of diabetic ketosis and bacterial cystitis during the study period. Additionally, the owners reported no adverse effects of *A. paniculata* supplementation throughout the study.

## 4 Discussion

Canine DM is a prevalent problem in veterinary medicine (Gilor et al., 2016). The application of plant-derived metabolites for the management of canine diabetes has been studied extensively (Russell et al., 2008). *Bixa orellana* extract was reported to decrease the blood glucose level by increasing peripheral glucose utilization in streptozotocin-induced diabetic dogs (Russell et al., 2008; Ogbu et al., 2013). *Gongronema latifolium* maintained the plasma glucose levels in alloxan-induced diabetic dogs by delaying stomach emptying (Ogbu et al., 2013). Additionally, numerous medicinal plants are reported to have the potential to improve the management of diabetes in humans (Bindu and Narendhirakannan, 2018). *A. paniculata*, a medicinal plant typically used as an anti-inflammatory and antimicrobial agent, has been studied for its possible impact on the management of DM in humans (Nugroho et al., 2012; Dai et al., 2019; Adiguna et al., 2021; Hossain et al., 2021). Andrographolide is a potential bioactive phytochemical in *A. paniculata*, which possesses antidiabetic properties (Nugroho et al., 2012; Ji et al., 2016; Islam, 2017; Naik et al., 2017; Mehta et al., 2021; Syukri et al., 2021). Plasma andrographolide was detected in dogs orally treated with *A. paniculata* tablets, indicating that andrographolide can be absorbed *via* the GI tract (Xu et al., 2015). However, it is important to be aware of the adverse effects of these natural products. Knowledge about the effectiveness and safety of *A. paniculata* in canine DM is limited. Therefore, the aim of this study was to investigate the effects of *A. paniculata* supplementation on the levels of glucose, fructosamine, inflammatory cytokines, and oxidative stress markers, and to determine the presence of any adverse effects in canine diabetes.

Initially, the animals were treated with 50 mg/kg of *A. paniculata* for 90 days, based on previous studies on rats, to determine the long-term efficacy and safety of the medicinal plant; however, no improvements were observed in the treatment group. Therefore, the dose and duration of *A. paniculata* supplementation were increased to 100 mg/kg/day for 180 days; however, no significant differences in blood glucose and fructosamine levels were observed in the diabetic dogs. The dose and frequency of insulin administration to the dogs were not adjusted during *A. paniculata* supplementation. However, the findings of this study were not in concordance with those reported in mice and rats (Yu et al., 2003; Akhtar et al., 2016; Chen et al., 2020; Jaiyesimi et al., 2020; Wediasari et al., 2020). Oral andrographolide and *A. paniculata* lowered the blood glucose levels of streptozotocin-induced diabetic rats in a dose-dependent manner (Yu et al., 2003; Wediasari et al., 2020). Furthermore, *A. paniculata* extract was reported to reduce hyperglycemia by inhibiting β-cell dysfunction in alloxan-induced diabetic rats (Jaiyesimi et al., 2020). An ethanolic extract of *A. paniculata* and andrographolide lowered the plasma glucose levels by enhancing the translocation of glucose-transporter-4 in insulin-resistant obese mice (Akhtar et al., 2016; Chen et al., 2020).

Hyperglycemia elevates the levels of inflammatory markers and promotes reactive oxygen species (ROS) formation, leading to several DM complications (Siddiqui et al., 2019). Furthermore, increased oxidative stress and inflammation could result in insulin resistance and reduced β cell function. These vicious cycles are linked to the pathogenesis of DM. In the current study, no changes in the plasma levels of IL-6 and TNF-α were observed in the *A. paniculata-*treated diabetic dogs. However, the extract has been reported to significantly reduce the levels of these markers in diabetic rats (Jaiyesimi et al., 2020). In another study, treatment with andrographolide suppressed cardiac inflammation *via* nuclear factor-κB (NF-κB) and reduced both cardiac fibrosis and cardiac hypertrophy in streptozotocin-induced diabetic mice (Liang et al., 2018). Similarly, andrographolide reportedly blocked the NF-κB signaling pathway induced by TNF-α in adipocytes, suggesting that it might reduce insulin resistance by modulating the insulin signaling pathway and improving glucose uptake (Jin et al., 2011; Chen et al., 2020). In the current study, no significant differences in the levels of the oxidative stress markers, including SOD and MDA were observed between the healthy and diabetic groups of dogs. This is contrary to previous reports, which indicated the beneficial effect of *A. paniculata* and andrographolide on DM complications *via* reductions in oxidative stress. For instance, andrographolide reduced the characteristics of diabetic nephropathy in murine glomerular mesangial cell lines by reducing the intracellular oxidative states. As considered, andrographolide renal hypertrophy and extracellular matrix accumulation in diabetic mice, as well as NADPH oxidase-1 (NOX-1) expression, ROS production, and proinflammatory cytokines (Lee et al., 2010; Ji et al., 2016). In addition, the cardioprotective effects of andrographolides in diabetic cardiomyopathy in mice have been associated with a decrease in ROS produced by NOX activation (Liang et al., 2018).

The etiology and type of diabetes differ across species, which might explain the variability in the responses to *A. paniculata* treatment. Insulin deficiency, the most common type of canine diabetes, is characterized by the autoimmune destruction of pancreatic β-cells, which leads to insufficient insulin production and glucotoxicity comparable to that seen in human type 1 diabetes (T1DM) (Nelson and Reusch, 2014; Gilor et al., 2016; O'Kell et al., 2017). Assuming dogs with clinical diabetes have end-stage T1DM may result in a lost potential to treat and reverse glucotoxicity (Gilor et al., 2016). This might explain why *A. paniculata* treatment did not improve the characteristic features of canine DM in the present study. Furthermore, the findings of the current study were inconsistent with those of other rodent studies, which reported lowered glucose levels and decreased DM complications after treatment with *A. paniculata* and andrographolide (Yu et al., 2003; Lee et al., 2010; Nugroho et al., 2012; Ji et al., 2016; Islam, 2017; Naik et al., 2017; Wediasari et al., 2020; Mehta et al., 2021; Syukri et al., 2021). However, the DM in rodents was chemically induced with streptozotocin or alloxan, which promotes cell death and decreases insulin production; this is different from the canine DM produced by chronic autoantibody destruction over a long period (Al-awar et al., 2016; Furman, 2021; O’Kell et al., 2022). *A. paniculata* treatment was reported to lead to beneficial outcomes in insulin-resistant obese mice, a model for type 2 diabetes (T2DM), which is not frequent in dogs (Catchpole et al., 2005; Akhtar et al., 2016; Heydemann, 2016; Chen et al., 2020).

Interspecies variations in pharmacokinetics, particularly the absorption process, may impact the response to *A. paniculata* treatment. Although oral *A. paniculata* tablets were absorbed *via* the GI tract in dogs, the information about the pharmacokinetics involved is currently lacking (Xu et al., 2015). Furthermore, variations in bioavailability across studies beyond species variations may be related to the use of various forms of *A. paniculata* (Loureiro Damasceno et al., 2022; Songvut et al., 2022). Andrographolide metabolism and clearance variations have been observed among species (Panossian et al., 2000; Zhao et al., 2013). DM involves complicated metabolic processes; hence, the parameters utilized to assess the impact of *A. paniculata* in this study, such as the blood glucose, fructosamine, cytokines (IL-6 and TNF-α), and oxidative stress indicators (SOD and MDA), may not sensitive enough. The pathogenesis and complications of DM, along with the treatment response, have been analyzed using proteomic patterns (Riaz, 2015; Pena et al., 2016; Kim et al., 2019). Candidate proteins which including alpha-1-antichymotrypsin, alpha-1-antitrypsin, apolipoprotein A-I (apoA-I), haptoglobin, retinol-binding protein 4, transthyretin, and zinc-alpha2-glycoprotein were shown to vary significantly between normal and prediabetes/diabetes in human patients (Riaz, 2015; Kim et al., 2019). Plasma proteomic analysis in canine DM showed a differential expression of alpha-2-HS glycoprotein, transthyretin, apolipoprotein A-I, and apolipoprotein A-IV compared with healthy dogs (Suemanotham et al., 2022). Plasma potential biomarkers such as apolipoprotein C-I, apoA-I, transthyretin, and cystatin C were purposed to predict the progression and response to the treatment in diabetic kidney disease (Riaz, 2015; Pena et al., 2016). Consequently, the effects of *A. paniculata* on canine diabetes should be evaluated using a proteomic approach and involving a wider variety of protein markers.

Confounding factors such as diet, activities, and environment were common limitations in this clinical study. Therefore, dogs that were fed a commercial diabetic diet, allowed to live indoors or within compounds close to their owners, and did not experience any changes in their environment before and during the study period, were included in the present study. The randomized, double-blind placebo control trial, considered as the gold standard method for medical research, was used in this study to mask the bias. Thus, we believe that, based on the strict inclusion and exclusion criteria and the robust study design, the results of this study should adequately represent the effect of *A. paniculata*, despite the clinical confounding factors.

Physical examinations were conducted, and the biochemical parameters of liver injury and kidney function were evaluated during the treatment periods. Additionally, adverse reactions such as weakness, vomiting, diarrhea, and allergy were monitored. No changes in physical and biochemical parameters, including ALT, ALP, BUN, and creatinine levels, were noted in all dogs supplemented with *A. paniculata* (dosages, 50 and 100 mg/kg/day for 90 and 180 days, respectively). Andrographolide was reported to exert beneficial effects by lowering the levels of urea, BUN, and creatinine in streptozotocin-induced diabetic rats (Xu et al., 2012). In another study, *A. paniculata* demonstrated protective effects against hepatic injury induced by paracetamol and ethanol in rodents (Nagalekshmi et al., 2011; Sivaraj et al., 2011; Mondal et al., 2022). Adverse effects, including nausea, vomiting, abdominal discomfort, dizziness, drowsiness, and urticaria, of *A. paniculata* extracts or pure metabolites, were observed in some human clinical studies (Thamlikitkul et al., 1991; Saxena et al., 2010; Jayakumar et al., 2013). Non-etheless, no adverse effects were observed in any of the treated dogs in the present study. The worsening of the clinical signs of DM, such as weight loss, polyphagia, polyuria, and polydipsia, was not encountered. These findings suggest that the dosages of *A. paniculata* administered were harmless within the trial durations specified in this investigation.

The supplementation of *A. paniculata* did not statistically improve the clinical signs and did not affect the DM parameters or the inflammatory and oxidative stress markers in the current study, which might be attributed to three main reasons. First, the doses and treatment period of *A. paniculata* supplementation were insufficient. Secondly, the pharmacokinetic variations among species and dosage forms of *A. paniculata* may impact the plasma concentrations of andrographolide, leading to variations in the pharmacological response (Zhao et al., 2013; Loureiro Damasceno et al., 2022; Songvut et al., 2022). Lastly, the biomarkers we used were not sensitive enough to identify the changes in the etiology and complications of DM. Therefore, protein profiling has been suggested to better understand the prognosis, development of complications, and treatment outcome prediction of DM (Chen and Gerszten, 2020; Araumi et al., 2021; Gummesson et al., 2021). However, unless the appropriate biomarkers can be evaluated, there is still a possibility that *A. paniculata* can contribute to delay the development and consequences of canine diabetes. Taken together, the findings of this study suggest that treatment with *A. paniculata* for 180 days did not affect the clinical parameters and levels of inflammatory cytokines and oxidative stress markers in canine DM. Additionally, no adverse effects were observed in the diabetic animals. These positive attributes demonstrate the tolerability of *A. paniculata* for long-term treatment in canine diabetes. Non-etheless, the relevance of other biomarkers such as apolipoprotein A-I (apoA-I), transthyretin, and alpha2-glycoprotein for observing the changes in the pathogenesis of DM must be explored further.

## Data Availability

The raw data supporting the conclusions of this article will be made available by the authors, without undue reservation.
