# Supplementary material for: Antidiabetic effects of Andrographis paniculata supplementation on biochemical parameters, inflammatory responses, and oxidative stress in canine diabetes
Source: Front Pharmacol. 2023 Feb 14;14:1077228. doi: 10.3389/fphar.2023.1077228 (PMC9971231; doi:10.3389/fphar.2023.1077228)
Supplement: Supplementary file 1 [file DataSheet2.PDF]

Supplementary material A

Table 1 Signalment, diet and husbandary history of diabetic dogs enrolled in the study

DM Dog	Protocol*	Breed	Gender	Age (year)	Weight (kg)	Diet	Living	Environmental change
1	1: treatment	Pug	Male	9	16.2	Commercial canine diabetic	Indoor	No
2	1: treatment	Poodle	Female	5	6	Commercial canine diabetic	Indoor	No
3	1: treatment	Poodle	Male	3	8.6	Commercial canine diabetic	In compound	No
4	1: treatment	Shih Tzu	Female	9	7.4	Commercial canine diabetic	Indoor	No
5	1: treatment	Pomeranian	Male	9	8.8	Commercial canine diabetic	Indoor	No
6	1: treatment	Labrador Retriever	Male	6	29	Commercial canine diabetic	In compound	No
7	1: placebo	West Highland White Terrier	Female	10	8	Commercial canine diabetic	Indoor	No
8	1: placebo	Chihuahua	Female	6	3.9	Commercial canine diabetic	indoor	No
9	1: placebo	Poodle	Female	8	5.5	Commercial canine diabetic	indoor	No
10	1: placebo	Beagle	Male	8	9	Commercial canine diabetic	In compound	No
11	1: placebo	Shih Tzu	Female	4	6.1	Commercial canine diabetic	indoor	No
12	1: placebo	Mixed breed	Male	7	15.3	Commercial canine diabetic	In compound	No
13	1: placebo	Golden retriever	Female	6	28	Commercial canine diabetic	In compound	No
14	2: treatment	Beagle	Male	5	10.4	Commercial canine diabetic	indoor	No
15	2: treatment	Poodle	Female	13	7.9	Commercial canine diabetic	indoor	No
16	2: treatment	Shih Tzu	Female	4	6	Commercial canine diabetic	In compound	No

17	2: treatment	Mixed breed	Male	7	18.6	Commercial canine diabetic	In compound	No
18	2: treatment	Pomeranian	Male	5	4.2	Commercial canine diabetic	indoor	No
19	2: treatment	Cocker Spaniel	Female	8	14.6	Commercial canine diabetic	indoor	No
20	2: placebo	Pomeranian	Male	6	3.9	Commercial canine diabetic	indoor	No
21	2: placebo	Mixed breed	Female	9	20	Commercial canine diabetic	In compound	No
22	2: placebo	Shih Tzu	Male	7	7.5	Commercial canine diabetic	indoor	No
23	2: placebo	Poodle	Female	11	7	Commercial canine diabetic	indoor	No

* Protocol 1: received *A. paniculata* extracted capsules 50 mg/kg/day (n=6) or placebo (n=7) for 90 days; Protocol 2: received *A. paniculata* extracted capsules 100 mg/kg/day (n=6) or placebo (n=4) for 180 days.
