# Supplementary material for: Antidiabetic effects of Andrographis paniculata supplementation on biochemical parameters, inflammatory responses, and oxidative stress in canine diabetes
Source: Front Pharmacol. 2023 Feb 14;14:1077228. doi: 10.3389/fphar.2023.1077228 (PMC9971231; doi:10.3389/fphar.2023.1077228)
Supplement: Supplementary file 2 [file DataSheet4.PDF]

Supplementary material B

Determination of andrographolide in the *Andrographis paniculata* (Burm.f.) Nees [Acanthaceae] (*A. paniculate*) capsule

1. Material and Method.

1.1 Sample Preparation

The *A. paniculata* powder (1.42 g) was soaked with absolute ethanol (30 mL) thrice. The supernatant was collected and evaporated to obtain the dried *A. paniculata* extract (0.08 g, %yield = 5.63) for further determination.

1.2 Quantitative Determination of Andrographolide Content in *Andrographis paniculata* Extract using High Performance Liquid Chromatography (HPLC)

A. paniculata extract was subjected to determine andrographolide content by HPLC using a C18 column (250 × 4.6 mm, 5 µm) (Agilent Technologies, Santa Clara, CA, USA). The chromatographic separation was carried out using a gradient system of mobile phase A (acetonitrile) and mobile phase B (water) with a total run time of 25 min for a detection and a flow rate of 1 mL/min. The gradient system used was 27% A in 0 min and 50% in 9 min, followed by 50% in the next 20 min and 27% in the next 25 min. *A. paniculata* extract (1 mg) was dissolved in 1 mL of methanol (MeOH) and injected into the column for the detection of andrographolide at 230 nm. The peak area and retention time of compounds found in the extract sample were evaluated in the comparison with the standard curve of standard andrographolide (0–500 µg/mL).

1.3 Qualitative Determination of Andrographolide in *Andrographis paniculata* Extract using Thin Layer Chromatography (TLC)

A. paniculata extract (1 mg) was dissolved in 1 mL of MeOH and was spotted (2 drops) from the bottom with an elution distance of 8 cm on a pre-coated silica gel aluminum plate 60F-254 (10 x 10 cm) (E. Merck, Germany). The *A. paniculata* extract was then subjected to TLC analysis alongside the reference standard of andrographolide, using an 85:15 mixture of chloroform and absolute ethanol and then visualized under UV 254 nm to ensure the presence of andrographolide in the sample.

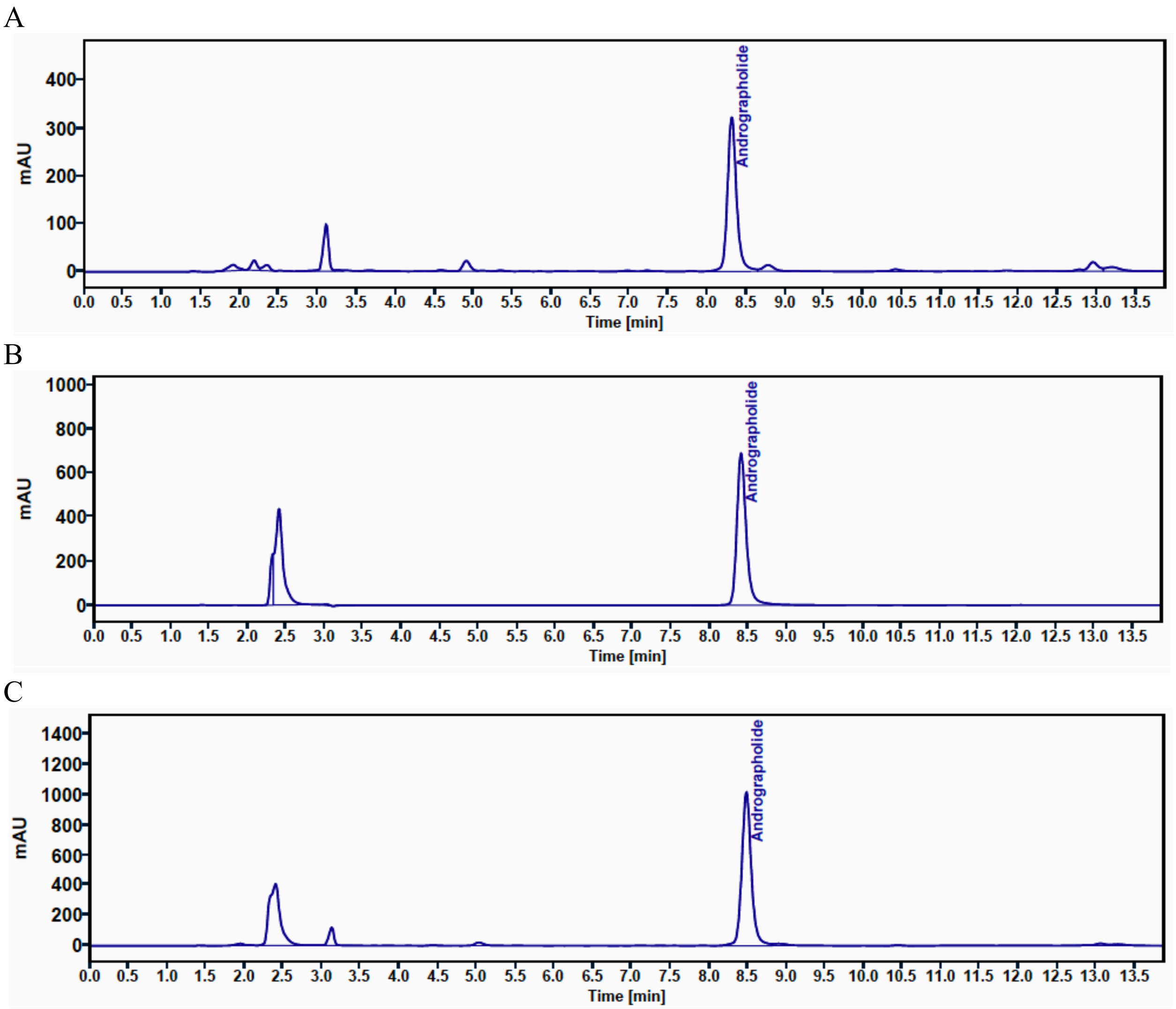

Figure 1. *A. paniculata* extract (A) and andrographolide (B) HPLC chromatograms. The mixture of *A. paniculata* extract and andrographolide (C). UV detection at 230 nm

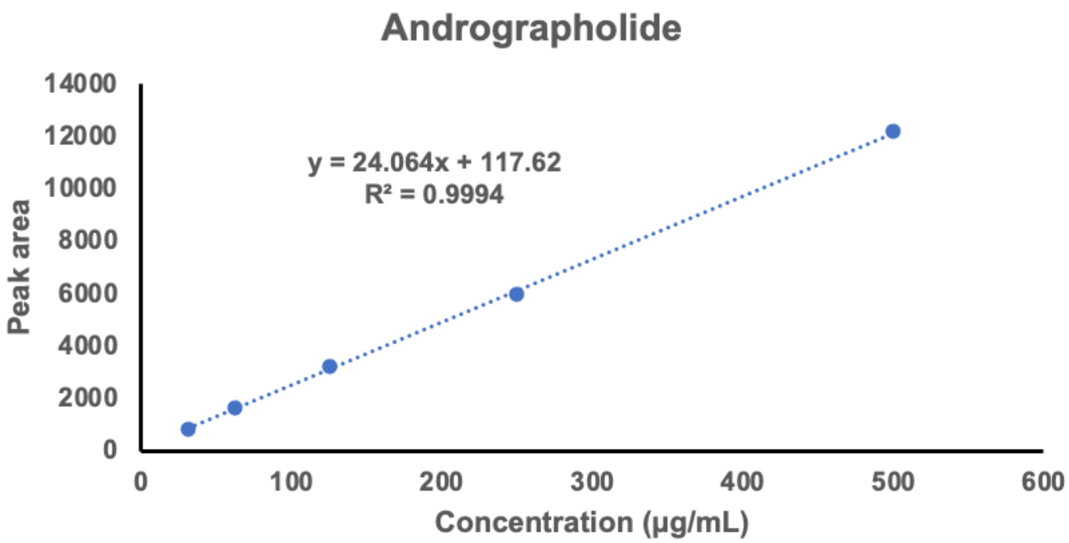

Figure 2. Standard curve of andrographolide

2. Results

2.1 Quantitative determination of Andrographolide Content in *Andrographis paniculata* Extract using High Performance Liquid Chromatography (HPLC)

Andrographolide content in *Andrographis paniculata* powder was determined by HPLC in comparison to the standard compound. The chromatogram of *Andrographis paniculata* extract was identified by comparing the retention time (RT) to that of the andrographolide standards. HPLC analysis of *A. paniculata* extract revealed a peak of the detected compound at RT = 8.446 (Figure 1A) which was compatible with that of the andrographolide standard (Figure 1B) (RT = 8.411). Additionally, the mixture of *A. paniculata* extract and andrographolide standard clearly demonstrated the perfect single peak of andrographolide at RT = 8.484 (Figure 1C). In the comparison with the standard curve (Figure 2), the amount of andrographolide in the *Andrographis paniculata* capsules was found to be 11.56 ± 8.1 mg/g dried plant powder or 4.57 mg/capsule (400 mg of *Andrographis paniculate* powder).

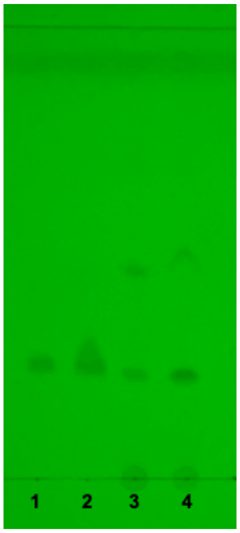

Figure 3. TLC chromatogram of *A. paniculata* extract under UV light at 254 nm. Adsorbent: silica gel GF254; Solvent system: $CHCl_3$: Absolute Ethanol = 85: 15. The standard of andrographolide at 500 and 1000 ug/mL was subjected to lane 1 and 2, respectively. Lane3 = *A. paniculata* extract and lane4 = a mixture of *A. paniculata* extract and standard andrographolide.

2.2 Qualitative Determination of Andrographolide in *Andrographis paniculata* Extract using Thin Layer Chromatography (TLC)

The chromatogram in Figure 3 shows a single mark of andrographolide in lane 1 and 2. The findings indicated that the trace *A. paniculata* extract in lane 3 has the same Rf value as andrographolide shown in lane 1 and 2. In addition, when mixing *A. paniculata* extract with standard andrographolide as shown in lane 4, the Rf value is similar to *A. paniculata* extract in lane 3 and andrographolide in lane 1 and 2. Therefore, the results confirmed that andrographolide was found predominantly in *A. paniculata* powder.
